# Vascularized Free Fibula Flap for Limb Salvage After Long Bone Tumor Resection in Pediatric Patients: A Single-Center Seven-Year Experience From a Developing Country

**DOI:** 10.7759/cureus.80187

**Published:** 2025-03-07

**Authors:** Fizzah Arif, Ansharah Mirza, Sobia Yasmeen, Mohammad Fazlur Rahman, Safdar Ali Shaikh

**Affiliations:** 1 Department of Plastic Surgery, Aga Khan University Hospital, Karachi, PAK; 2 Department of Plastic Surgery, Civil Hospital Karachi, Karachi, PAK

**Keywords:** free fibula flap, limb reconstruction surgery, limb-salvage, microsurgery, pediatric bone sarcoma

## Abstract

Background

Pediatric long bone sarcomas pose significant challenges, requiring a delicate balance between oncological control and limb preservation. Limb salvage surgery has emerged as a preferred approach, with vascularized free fibula flap reconstruction showing promise in achieving favorable outcomes. The objective of this study was to evaluate the clinical outcomes of limb salvage surgery, including complication rates, functional outcomes, long-term survival, and disease recurrence.

Method

A retrospective analysis was conducted on pediatric patients undergoing limb salvage surgery for long bone sarcomas using vascularized free fibula flaps. Data from a single center's experience over a seven-year period were analyzed, including patient demographics, tumor characteristics, surgical techniques, complications, and functional outcomes. Follow-up assessments were performed regularly to monitor bone healing, disease recurrence, and functional recovery.

Results

Fifteen pediatric patients underwent limb salvage surgery with vascularized free fibula flap reconstruction, with a focus on 10 cases meeting inclusion criteria. Osteosarcoma was the most common histological diagnosis (seven, 70%), predominantly affecting the lower extremities (six, 60%). Surgical procedures varied based on tumor location, with most reconstructions utilizing osteocutaneous fibula flaps (eight, 80%). The flap survival rate was 10 (100%), with no instances of flap failure. Immediate complications occurred in three (30%) of cases, primarily surgical site infections, while one patient experienced delayed non-union and fracture requiring additional surgery. Functional outcomes were generally favorable, with seven (70%) of patients retaining functional limbs during follow-up.

Conclusion

In conclusion, our study demonstrates that vascularized free fibula flap reconstruction in pediatric long bone sarcomas can achieve high flap survival and acceptable short-term functional outcomes. Our series supports the technical feasibility of this approach, and delayed complications such as non-union and metastasis underscore the need for careful long-term surveillance and further prospective studies.

## Introduction

Osteosarcoma and Ewing's sarcoma are the most prevalent kinds of sarcomas affecting the bones in children [1]. Pediatric long bone tumor management is extremely difficult to maintain the critical balance between curing the cancer and preserving limb function. These tumors are difficult to treat and require multidisciplinary management [2]. Complete surgical excision of the tumor is the main determinant affecting prognosis [1,2].

Sarcomas of the extremities have traditionally been treated mostly through amputation. However, limb salvage surgery, which aims to maintain normal limb function and anatomical integrity, has grown more popular due to breakthroughs in neoadjuvant and adjuvant chemotherapy [2].

Among the surgical approaches, the utilization of the free fibula flap has emerged as a promising reconstructive option [1,2]. Avascularized bone grafting, vascularized bone transfers, transposition of fibula, and endoprostheses can be employed for bony reconstruction. Endoprostheses had higher rates of infection and lengthening mechanism failure than biologic structures and hindered physiological growth of bone, although being less invasive [3].

Depending on the size of the bone defect, different reconstruction techniques are favored, with vascularized bone flaps being preferred for larger lesions. Vascularized bone flaps provide speedier recovery to full weight-bearing and ambulation, which is critical for patients receiving chemotherapy [1-3], higher rates of bony union, and reduced infection rates. In a retrospective cohort study conducted by Karami et al., the authors investigated the fibula flap as a reconstructive option for long bone tumors in pediatric patients. Their findings demonstrated satisfactory functional outcomes and fewer complications, supporting the efficacy of the free fibula flap in this population [4]. The longitudinal growth of pediatric patients can be maintained with physeal transfer in fibula flap [2].

Furthermore, Borthakur et al. presented their experience with free fibula reconstruction for bone tumors involving the humerus. Their study highlighted the successful use of the free fibula flap in restoring upper limb function and achieving long-term oncologic control [5]. The benefits of ample bone stock, similar survival rate to adults, lesser complication rate, dependable vascularity, and improved functional outcomes have made the free fibula flap a popular alternative for reconstruction [4,6].

This retrospective research aims to present our experience with the fibula flap for both upper and lower extremities reconstruction in pediatric bone tumors. The primary objective of this study was to evaluate the clinical outcomes of limb salvage surgery using vascularized free fibula flap reconstruction in pediatric patients with long bone sarcomas from a developing country. Secondary objectives included assessing complication rates, functional outcomes, long-term survival, and disease recurrence.

## Materials and methods

This retrospective cohort study was conducted using data from the Aga Khan University Hospital, Karachi Electronic Health Record (EHR), spanning January 2017 to May 2023. This study was exempted from Institutional Review Board approval. All surgeries were performed by the same oncologic orthopedic surgeon, and reconstructions by the same plastic surgeon, ensuring uniformity in surgical technique and post-operative care.

The study focused on pediatric patients aged between one and 18 years and included those who had undergone oncologic resections for long bone sarcomas (specifically humerus, femur, and tibia) with immediate reconstruction using vascularized free fibula flaps. Exclusion criteria comprised reconstructions with non-vascularized fibula grafts, benign limb conditions like chronic osteomyelitis or benign tumors, and bone defects due to trauma. Consecutive sampling was done, where all eligible cases meeting the inclusion criteria during the study period were enrolled without selection bias.

Eligible patients' data, including demographics, tumor histopathology, affected bone, lesion size, vascular anastomosis details, follow-up duration, complications, and any additional surgeries, were retrieved from electronic medical records.

Neoadjuvant chemotherapy was administered four weeks prior to surgery to reduce tumor size and improve resectability. After sterile measures were taken and antibiotic prophylaxis administered, oncologic resections were performed with wide tumor-free margins confirmed intraoperatively. The fibula was harvested in a standard pattern, with its vascular pedicle (peroneal artery and veins), and transferred to the defect site. Microsurgical vascular anastomoses were performed to ensure adequate perfusion of the graft. Stability was achieved using internal fixation devices (plates and screws). Following wound healing, adjuvant chemotherapy was initiated, with or without radiotherapy, as per the recommendations of the multidisciplinary tumor board. Flap monitoring was done based on clinical signs of the color of flap, bleeding on pin prick, turgor, and temperature. In buried flaps, the assessment was done based on signs of infection and portable Doppler.

Patients underwent follow-up assessments every three months, primarily conducted by the orthopedic surgeon. Extremity radiographs were taken during these visits to monitor bone healing and union. Additionally, chest X-rays and thoracic CT scans were performed every six months for the first two years post-resection and annually thereafter to check for disease recurrence or metastasis. 

Complications were categorized as immediate (e.g., flap issues, arterial or venous problems, congestion, flap loss, hematoma, seroma, infection) or late (nonunion, fractures). Radiological assessments determined bone union, defined by bridging callus on three of four cortices on plain radiographs or absence of an osteotomy line at graft ends.

Patient outcomes, including survival, disease recurrence, and limb preservation, were documented during final follow-up, with all patients having at least one year of clinical monitoring. Functional outcomes were evaluated using Mankin functional outcome scores [7] and Musculoskeletal Tumor Society Rating Scale-93 (MSTS-93) [8]. Limb salvage failure in the Mankin score was indicated by amputation, recurrence, distant metastasis, or death.

Data analysis was performed using the IBM Statistical Package for the Social Sciences Version 23 (IBM Corp., Armonk, NY). Frequencies and percentages were calculated for categorical variables such as sex, tumor histopathology, complications, site of involved limb, complication, etc. Descriptive statistics, including range/means and standard deviations, were used to summarize continuous variables such as patient age, follow-up duration, and lesion size.

## Results

**Figure 1:**
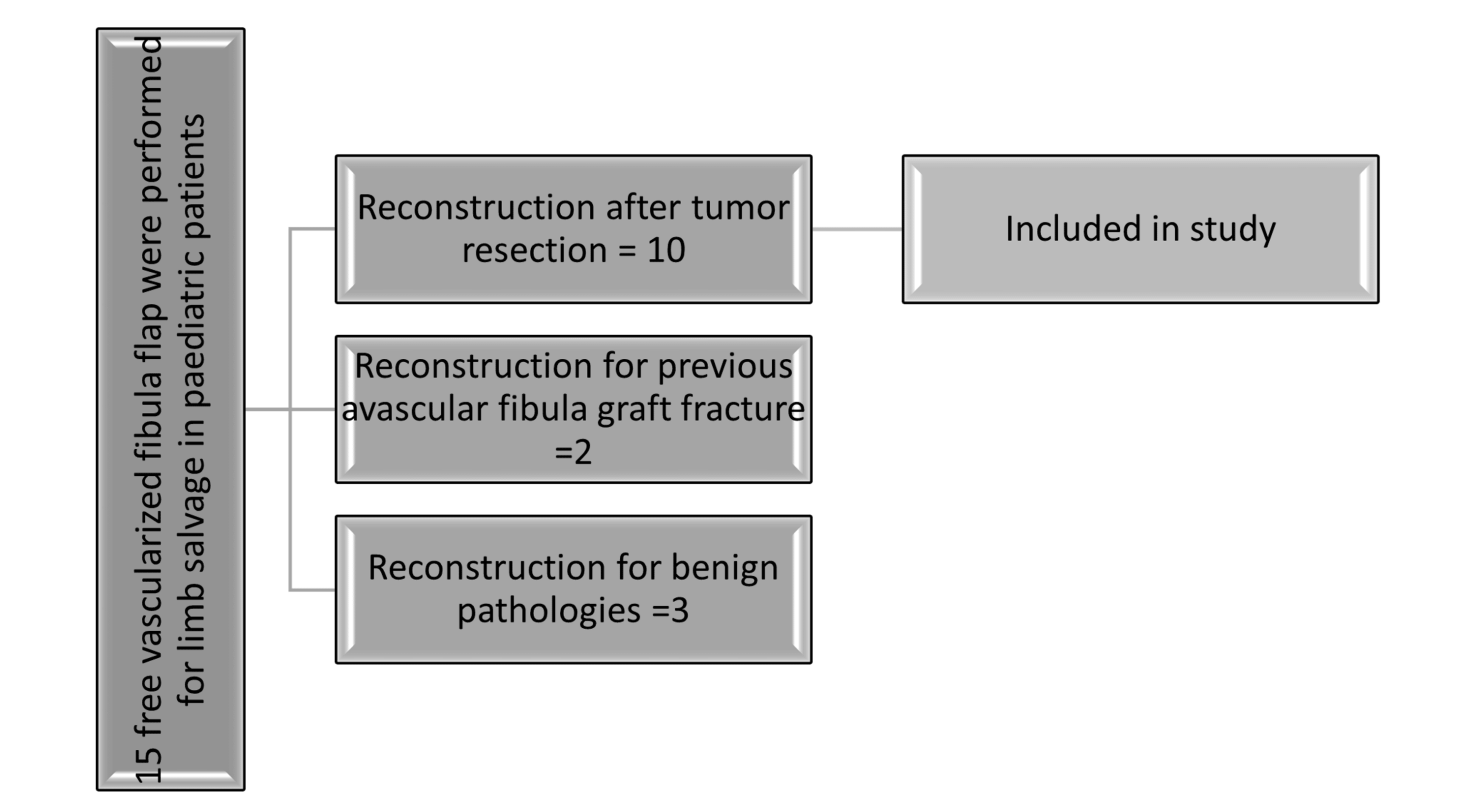
Flowchart illustrating the patient selection process undergoing free vascularized fibula flap (Designed by: Fizzah Arif).

We discovered 15 instances of utilizing a free fibula flap to salvage limbs in pediatric patients. Out of these cases, 10 patients who had undergone surgical removal of a long bone sarcoma were selected for the research. These patients had their limbs reconstructed using a vascularized fibula flap subsequent to the excision of a cancerous bone tumor (Figure 1). 

**Figure 2:**
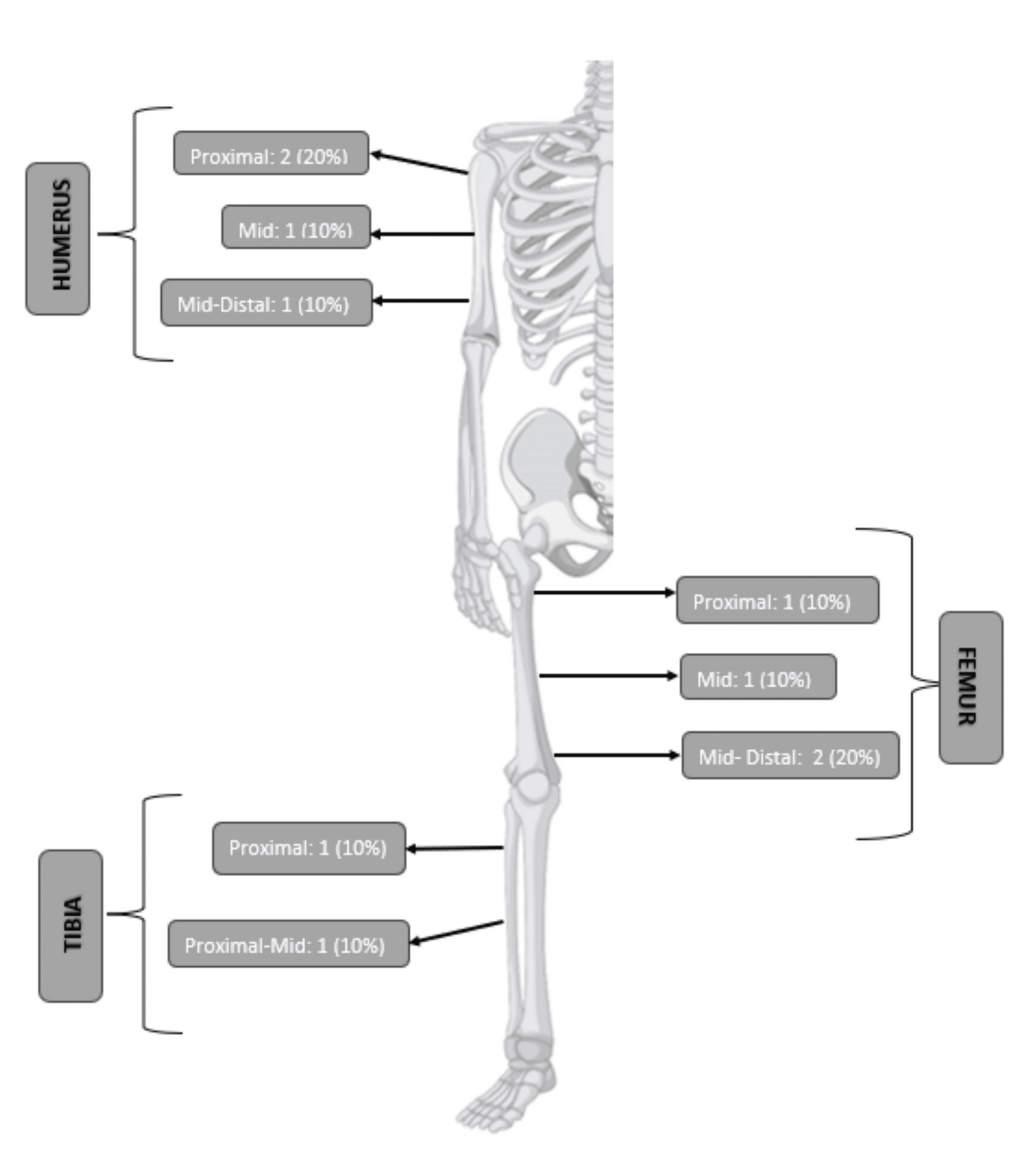
Bone and site involved by tumor (Designed by: Fizzah Arif).

There were three females (30%) and seven males (70%). The mean age was 9.2 ± 3.5 years. The pathologic diagnosis was osteosarcoma in seven (70%) patients and Ewing’s sarcoma in three (30 %) patients. Six (60%) patients had right-sided limb involvement, and four (40%) patients had left-sided lesions. Sarcomas were located in the upper limb in four (40 %) and in the lower extremity in six (60 %). Humerus was involved in four (40%) cases, femur in four (40%) patients, and tibia in two (20%) patients (Figure 2). Table 1 demonstrates details of patients and tumor characteristics.

**Table 1:** Patient demographics and tumor characteristics

Case no.	Age	Gender	Histological diagnosis	Side	Involved bone	Site	Local recurrence	Metastasis	Alive
1	15	F	Osteosarcoma	Left	Femur	Mid, distal	No	No	Yes
2	8	M	Osteosarcoma	Left	Tibia	Proximal	No	Pulmonary (radical surgery)	Yes
3	6	M	Osteosarcoma	Left	Humerus	Mid	No	No	Yes
4	15	M	Osteosarcoma	Right	Humerus	Proximal	Yes	Pulmonary	Yes
5	8	M	Osteosarcoma	Right	Humerus	Proximal	No	No	Yes
6	11	M	Osteosarcoma	Right	Femur	Mid, distal	No	Pulmonary	No
7	11	F	Ewing’s sarcoma	Right	Femur	Proximal	No	Bones	No
8	8	M	Ewing’s sarcoma	Right	Tibia	Proximal, mid	No	No	Yes
9	4	M	Osteosarcoma	Left	Femur	Mid	No	No	Yes
10	6	F	Ewing’s sarcoma	Right	Humerus	Mid, distal	No	No	Yes

All patients underwent the transfer of a vascularized fibula flap to address bone defects in their limbs. The average length of the fibula harvested for this purpose was 18.7 cm, ranging from 14 to 23 cm. In two (20%) cases, the fibula head was also included in the reconstruction. The flaps were harvested using the standard method, relying on the peroneal vessels. In six (60%) cases, avascular fibular bone grafts or autoclaved bone were utilized. Notably, one case involved a unique approach where the bone graft was obtained from the patient's mother, who was the youngest recipient. Of the flaps, eight (80%) were osteocutaneous, and two (20%) were osseous. There was no requirement for additional flaps to cover soft tissue defects.

Regarding the recipient artery, it varied based on the location of the defect. In cases involving the humerus, a branch of the brachial artery was utilized (four, 40%), while the anterior tibial artery was used for tibia reconstruction (two, 20%). For femur reconstruction, branches of either the superficial femoral artery (two, 20%) or the profunda femoris artery (two, 20%) were employed. All arterial and vascular anastomoses were hand-sewn, either in an end-to-end or end-to-side manner, without resorting to vein grafts or arteriovenous loops.

**Figure 3:**
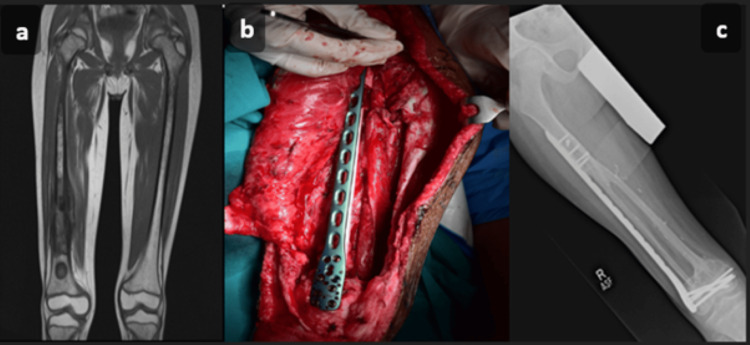
(a) Eleven-year-old with right femur osteosarcoma seen on magnetic resonance imaging (pre-operative). (b) Right femur bone defect after insetting of free fibula flap (intra-operative). (c) Plain radiograph with the fibula set in place (immediate post-operative) (Photography and Designed by: Safdar Ali Sheikh).

**Figure 4:**
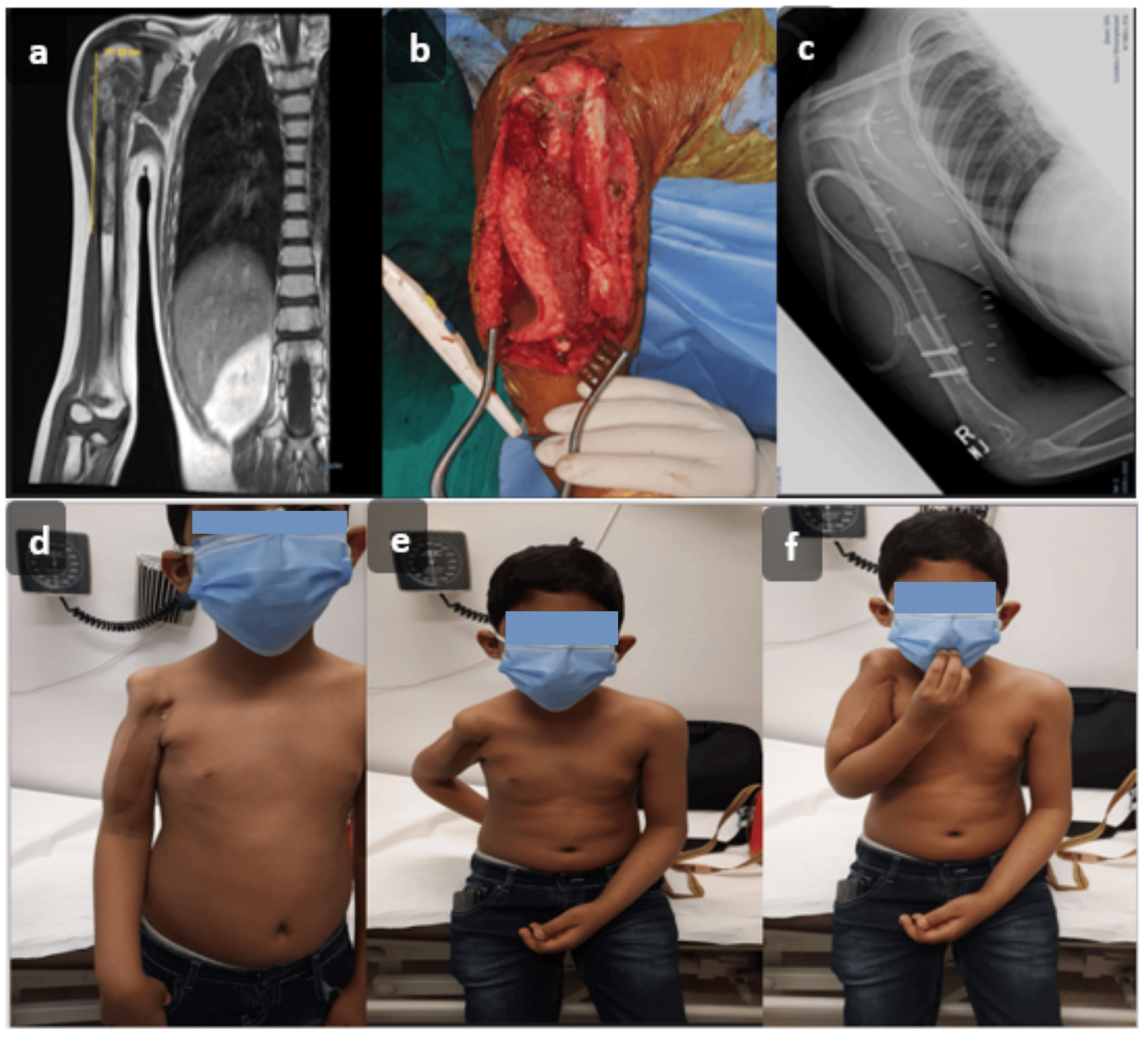
(a) Eight-year-old child with right humerus osteosarcoma seen on magnetic resonance imaging (pre-operative). (b) Right humeral bone defect after placement of free fibula flap with head (blue star). (c) Post-operative plain radiograph with the fibula in place. (d-f) Images showing function of upper limb (Photography and Designed by: Safdar Ali Sheikh).

The method of bony fixation was mostly internal fixation (Figure 3 and Figure 4). Locking compression plates were predominantly used for lower extremity reconstructions (five, 50%) and in some upper limb cases (two, 20%). Screws were employed in two cases (20%), one each for upper and lower limb reconstruction, while a reconstruction plate was used for humerus reconstruction in one case (10%). The average hospital stay was 5.2 days, ranging from three to nine days. Operative details are summarized in Table 2.

**Table 2:** Operative details LCP = locking compression plate; LOS = length of stay; OC = osteocutaneous; O = osseous

Case no.	Involved bone	Fibula length (cm)	Fibula with head	Accessory bone graft used	Recipient vessels	Anastomosis	Mode of fixation	Skin island	LOS (days)
1	Femur	23 cm	No	No	Femoral artery	End to side	LCP	OC	6
2	Tibia	20 cm	No	Yes	Anterior tibial artery	End to side	LCP	OC	5
3	Humerus	17 cm	No	Yes	Brachial artery	End to side	LCP	OC	9
4	Humerus	22 cm	No	No	Branch of brachial artery	End to end	Recon plate	OC	6
5	Humerus	18 cm	Yes	No	Brachial artery	End to side	Cortical screws	OC	3
6	Femur	18 cm	No	Yes	Branch of profunda femoris artery	End to end	LCP	O	5
7	Femur	22 cm	Yes	Yes	Femoral artery	End to side	LCP	OC	6
8	Tibia	15 cm	No	No	Anterior tibial artery	End to side	Cancellous and cortical screws	OC	3
9	Femur	14 cm	No	Yes (from mother)	Branch of profunda femoris artery	End to end	LCP	OC	4
10	Humerus	18 cm	No	Yes	Brachial artery	End to side	LCP	O	5

Complications

The survival rate of flaps was 10 (100%), with no instances of failure. There were no complications during surgery. However, postoperatively, three (30%) of patients encountered complications categorized as immediate or delayed. All three patients experienced immediate issues, with one patient also experiencing a delayed complication. There were no occurrences of arterial thrombosis or venous congestion; thus, no urgent re-explorations were warranted.

Among immediate complications, three (30%) of cases suffered from surgical site infections at the flap recipient site. Two (20%) required surgical wound debridement in the operating theater, while the remaining one (10%) was managed conservatively with antibiotics and daily dressings, resulting in resolution. One patient, who initially experienced a surgical site infection, later encountered a late complication involving non-union and fracture (one, 10%) of the fibula flap, necessitating open reduction and internal fixation (ORIF) with dynamic compression plate (DCP). Subsequently, a second free fibula flap was required due to the previous fracture and broken implant. Fortunately, there were no complications at the donor site. Table 3 delineates immediate and delayed complications, further stratified by donor versus recipient site.

**Table 3:** Complications by onset (immediate vs. delayed) and site (donor vs. recipient)

S. no.			Complications	n (%)
1	Intraoperative		None	0
2	Post-operative			
	Immediate	Donor	None	0
		Recipient	Surgical site wound infection	3 (30%)
	Delayed	Donor	None	0
		Recipient	Nonunion and fibula fracture	1 (10%)

Table 4 presents the operative revisions for each of the three patients.

**Table 4:** List of secondary/revision surgeries performed DCP = dynamic compression plate; ORIF = open reduction and internal fixation; VFFF = vascularized free fibula flap

Case no.	Secondary procedures
1	ORIF + DCP for nonunion
	Free fibula flap (second time) for fractured previous VFFF
2	Surgical site wound debridement (recipient)
	Diagnostic thoracoscopy + wedge resection of right upper lung lobe
4	Surgical site wound debridement (recipient)
	Right shoulder disarticulation
3,5,6,7,8,9,10	None

In total, three patients (30%) underwent six additional surgeries. On average, patients requiring surgical intervention underwent two surgeries each. Seven (70%) patients did not require any additional surgical procedures.

These secondary procedures encompassed various treatments, including addressing nonunion and fracture in one patient, performing wound debridement in two patients, and shoulder disarticulation in one patient. Additionally, one patient underwent thoracoscopy and wedge resection to address a metastatic lesion in the upper lobe of the right lung.

Follow-up, oncologic, and functional outcomes

The average follow-up duration was 23.8 months, ranging from four to 70 months (Table 5). Of the patients, four (40%) developed metastasis, primarily to the lungs (three, 30%), with one case (10%) in the pelvis and vertebra. However, 60% of patients remained disease-free. Among them, seven (70%) retained a functionally salvaged limb during follow-up, with one patient (10%) requiring shoulder disarticulation due to local recurrence and pulmonary metastasis at the latest follow-up. Two patients (20%) succumbed to the condition. The overall disease-free rates at one and two years were 10 (100%) and five (50%), respectively. Additionally, 40% of patients experienced disease recurrence or metastasis at an average of 19.75 months after limb salvage surgery. These details are summarized in Table 1.

**Table 5:** Survival and functional outcomes MSTS = Musculoskeletal Tumor Society Rating Scale-93

Case no.	Follow-up survival after surgery (months)	MSTS-93 score		Mankin functional outcome rating	
		Upper limb	Lower limb	Upper limb	Lower limb
1	27 months	-	25	-	Excellent
2	24 months	-	23	-	Failure (metastasis)
3	12 months	27	-	Excellent	-
4	12 months	None	-	Failure (disarticulation)	-
5	51 months	22	-	Good	-
6	15 months	-	None	-	Failure (death)
7	18 months	-	None	-	Failure (death)
8	70 months	-	25	-	Excellent
9	Five months	-	27	-	Excellent
10	Four months	21	-	Good	-

Functional outcomes are mentioned below for each case in Table 5. The Mankin functional outcome score (Appendix 1) and the MSTS-93 (Appendix 2 and Appendix 3) were used for outcome measurement. A Mankin functional outcome score, classified as excellent, was achieved in four (40%) of patients, with two (20%) classified as good. However, reconstruction failures occurred in four (40%) of patients, including metastasis in 1 (10%), amputation in 1 (10%), and death due to disease progression in 2 (20%). Following reconstruction, the average MSTS-93 rating for the seven patients was 24.28, equating to 80.93%. Lower extremity reconstructions demonstrated slightly higher MSTS-93 scores, 83.3% (ranging from 76.7% to 90%), whereas upper extremity reconstructions had an average score of 77.8% (ranging from 70% to 90%).

The average ratings (with ranges) for the variables common to all patients (n = 7) were as follows: pain (5, 5-5), function (4, 3-5), and emotional acceptance (4.2, 4-5). For the factors pertinent to upper extremity reconstruction (n = 3), the average scores (with range) were: hand position (3.7, 3-5), manual dexterity (3.7, 3-5), and lifting ability (3, 3-3). Similarly, for lower extremity reconstruction (n = 4), the average scores (with range) were: support (4, 4-4), walking ability (3.7, 3-4), and gait (3.7, 3-4).

## Discussion

Pediatric extremity bone sarcomas manifest with diverse demographic, histopathologic, and anatomical characteristics. In comparing our study's findings with those reported in the literature, notable similarities and divergences emerge, shedding light on the heterogeneity within pediatric extremity bone sarcomas.

In our study cohort, males were predominant, constituting 70% of the sample. The mean age of participants was 9.2 years, ranging from four to 15 years. This aligns closely with the average age of 10 years reported by Adam et al. [9] and 9.3 years reported by Ruiz-Moya et al. [10]. Regarding pathologic diagnoses, osteosarcoma accounted for most of the cases (70%), with Ewing’s Sarcoma being less prevalent (30%). This distribution contrasts with the findings in other study populations [10], where Ewing sarcoma predominated (59.26%), followed by osteosarcoma (40.74%). Analysis of the anatomic distribution of sarcomas in our study revealed a noteworthy balance between the upper and lower extremities (40% and 60%, respectively). This stands in contrast to the findings of McCullough et al. [11] and Adam et al. [9], where a significant majority of sarcomas were located in the lower extremities, 68.9% and 60%, respectively. Our study revealed that the majority of cases involved the humerus, femur, and tibia. This trend aligns with observations from another study, although variations in the frequency of involvement were noted; for instance, the femur was most involved in one study [10], whereas the tibia was predominant in others [11,12].

Our analysis revealed an average length of the fibula used for reconstruction to be 18.7 cm, with a range of 14 to 23 cm. This finding is consistent with observations reported by other surgeons, who have documented a mean harvested fibula length of 18 cm [9], a mean reconstructed length of 13.37 cm [10], and 12.87 cm (7-24 cm) [11]. The inclusion of the fibula head in a subset of cases underscores its versatility in addressing specific anatomical requirements, particularly in the reconstruction of the femoral, humeral, and distal radius head [10].

Noteworthy variations emerge in the selection of recipient vessels across different reconstructions. In our cohort, the choice of recipient artery intricately corresponded with the anatomical site being addressed: the brachial artery for humerus, the anterior tibial artery for tibia, and the superficial or profunda femoral artery for femur. The absence of vein grafts or arteriovenous loops, as uniformly noted across our study and Karami et al. study, underscores the reliability of pedicle length and efficacy of anastomosis planning in pediatric fibular reconstruction [4].

Numerous innovations have been developed to enhance limb strength using inset free fibula flaps. Vascularized free fibula flap (VFFF) can be used as a rescuing option for limb salvage in cases of non-union/fractures of allograft intercalary reconstructions [13]. Capanna [14] initially combined allografts with autologous fibular flaps for lower limb reconstruction post-tumor resections. While a vascularized free fibula flap lacks early ambulation strength, it promotes vascularity for optimal healing. Intercalary allografts provide mechanical stability crucial for weight bearing but can lead to delayed union, non-union, or fractures. The vascularized fibular graft (VFG) hypertrophies rapidly, but until that occurs, the allograft provides the necessary mechanical strength for early ambulation [15], achieving original tibial size in 90% of surviving patients during tibial reconstruction [16]. Thus, a combined approach proves advantageous. When using a VFG for femoral reconstruction, its ability to withstand body weight is paramount. To address this, Toh et al. introduced transplanting a folded fibular graft on a vascular pedicle in 1988 [17]. Another method involves inserting the diaphysis of a vascularized free fibula flap into a cadaver allograft's medullary canal to enhance strength [18]. Additionally, a case report describes vertically aligning bilateral free VFGs within a femoral allograft's medullary canal [19]. In our study, all fibulas were inset within the defect in a single barrel configuration. Pediatric patients (n = 6) received avascular fibular bone grafts or autoclaved bone, while in one unique case, the youngest patient received a bone graft from their mother.

Notably, none of our patients necessitated re-exploration or anastomosis revision, or additional flaps for soft tissue coverage. However, our observed overall immediate complication rate of 30%, affecting three out of 10 patients, prompted surgical wound debridement in two cases, while conservative management sufficed for one. In contrast, a starkly higher overall complication rate of 74.07%, 64%, and 53% was documented in a study by Ruiz-Moya et al. [10], Claxton et al. [20], and Edward et al. [21] with a significant portion of 32%, 39%, and 40%, requiring secondary or revision surgeries, respectively, predominantly at the recipient site. Common reported complications across various studies were fibula fracture [9,10,20-25], local wound infection [8,9,20-22,26], wound dehiscence [8,9,20,26], amputation [10,20,22], delayed union [10,20-22,24], fixation failure/hardware loosening [10,20,22], extensor hallucis palsy [9,22], ulnar impaction, compartment syndrome, hematoma [26], donor site ankle deformity/instability [26-28], claw toes [20,28], and paresis of the common fibular nerve [9]. Peroneal nerve deficits were seen in all patients with epiphyseal transfers [29].

Primary union occurs on average at 8.7 months [15] or on average at 9 ± 8 months [20], with another study reporting a 75% first-time union rate at 10 months [25] postoperatively. Female gender was associated with failure of primary union (p = 0.03), whereas age, tumor location above the elbow, radiotherapy, and adjuvant chemotherapy were not associated with failure to achieve primary union [20]. Also, the length of the graft has no significant correlation to the rate of union [15].

A study [10] reported an 18.52% metastasis rate and two deaths over a 44.33-month mean follow-up, while others [24] noted recurrence and two mortalities over 48.7 months. In our cohort, 40% of patients had metastasis, and two deaths were observed at 23.8-month average follow-up; 60% of our patients retained functionally salvaged limbs during follow-up. Comparative analyses with other studies [10,21] revealed higher rates of limb salvage success at 96.29% and 97%, respectively.

Comparative analysis using the MSTS-93 rating revealed a mean score of 24.28 (80.93%) in our study, closely aligned with findings from studies [20,22,24,30], where vascularized fibula flaps achieved a mean MSTS score of 82%, 82.5%, and 85.4%, respectively, emphasizing favorable functional outcomes. A review spanning 17 years reported MSTS scores of 85% in upper limbs and 93% in lower limbs with VFFF for limb salvage [12].

In our study, 40% of patients recorded excellent Mankin functional outcomes, and 20% had good outcomes. Another study reported good to excellent outcomes in 71%, with 11% experiencing reconstruction failures leading to distant metastases and 7% undergoing amputation for local recurrence [20]. The follow-up period in our study may not be sufficient to fully capture late-term complications or functional decline.

Our study reported disease-free survival rates of 100% at one year and 50% at two years. Unfortunately, 20% of our patients experienced disease progression leading to mortality within two years post-surgery. Delayed chemotherapy due to wound-related issues may have contributed to pulmonary metastasis in two of our patients. However, in patients with distant metastasis, survival rates at two, five, 10, and 15 years were 96%, 83%, 83%, and 55%, respectively [20]. Estimated five-year overall survival was 72%, and disease-free survival was 64% [24]. In another study, 40% of patients experienced disease recurrence/metastasis at an average of 19.75 months post-limb salvage surgery, contrasting with 93% disease-free survival at two years in a different study, with recurrence occurring at a mean of 4 ± 5 years post-surgery [20].

This study contributes insights into the interplay of reconstruction outcomes, disease progression, and functional recovery in pediatric extremity bone sarcoma cases using free vascularized fibula flaps. A few of the limitations include retrospective design, small sample size but comparable to previously published literature [4,16,26], short follow-up duration, lack of subgroup analyses, particularly comparing upper and lower extremity reconstructions, due to small sample size and lack of statistical power and detailed data on the exact bone defect sizes for each case were not systematically recorded at the time of surgery.

Future multi-center, prospective, and comparative studies with longer follow-up durations to better understand the long-term impact on children and further advance the field of pediatric limb salvage surgery.

## Conclusions

In conclusion, this retrospective study highlights that vascularized free fibula flap reconstruction in pediatric long bone sarcomas can achieve high flap survival and acceptable short-term functional outcomes. While our series supports the technical feasibility of this approach, the absence of comparative data limits claims regarding its versatility, significant incidence of metastasis/death, and delayed complications such as non-union and metastasis underscore the need for careful long-term surveillance and further prospective studies.

## Appendices

Appendix 1 

**Table 6:** Mankin functional score Source: [7]

	Grades	
1	Excellent	Patients were considered excellent when they had no evidence of local recurrence or metastasis, had normal function of the part, and were able to return to their normal job and home activities without significant limitations. For lower extremity patients to qualify for excellent they had to walk without a limp. All were free of pain, and many have engaged in athletic activities.
2	Good	Patients classified as good had no evidence of tumor but were moderately restricted in activities and had some limitation of function. All in this group returned to work activities and were free of pain but were unable to participate in some of the more active life activities, such as athletics.
3	Fair	Patients placed in the fair category showed no evidence of disease but showed fairly marked limitation of function. A brace or support was necessary for function. Although many of these patients were restored to preoperative work circumstances, all had restrictions on activities, and some had mild to moderate pain.
4	Failure	The failure group included two types of patients: those in whom the limb had to be amputated or the implanted part removed and those who died as a result of recurrent or metastatic tumors.

Appendix 2 

**Table 7:** Musculoskeletal tumor society scoring system for lower limb functionality Source: [8]

Score	Pain	Function	Emotional	Supports	Walking	Gait
5	No pain	No restriction	Enthused	None	Unlimited	Normal
4	Intermediate	Intermediate	Intermediate	Intermediate	Intermediate	Intermediate
3	Modest	Recreational restriction	Satisfied	Brace	Limited	Minor cosmetic
2	Intermediate	Intermediate	Intermediate	Intermediate	Intermediate	Intermediate
1	Moderate	Partial restriction	Accepts	One cane or crutch	Inside only	Major cosmetic
0	Severe disabling	Total restriction	Dislikes	Two cane or crutch	Not Independent	Major handicap

Appendix 3

**Table 8:** Musculoskeletal tumor society scoring system for upper limb functionality Source: [8]

Score	Pain	Function	Emotional	Hand positioning	Manual dexterity	Lifting ability
5	No pain	No restriction	Enthused	Unlimited	No limitations	Normal load
4	Intermediate	Intermediate	Intermediate	Intermediate	Intermediate	Intermediate
3	Modest	Recreational restriction	Satisfied	Not above shoulder	Loss of fine movements	Limited (minor load)
2	Intermediate	Intermediate	Intermediate	Intermediate	Intermediate	Intermediate
1	Moderate	Partial occupational restriction	Accepts	Not above waist	Cannot pinch	Helping only (cannot overcome gravity)
0	Severe disabling	Total occupational restriction	Dislikes	None	Cannot grasp	Cannot move
